# End-of-life decision-making in the ICU: perspectives on who “should” decide and influencing factors

**DOI:** 10.1192/j.eurpsy.2025.1541

**Published:** 2025-08-26

**Authors:** S. Georgakis, E. Dragioti, G. Papathanakos, M. Gouva, V. Koulouras

**Affiliations:** 1 Research Laboratory of Patient, Family & Health Professional Psychology, Department of Nursing, School of Health Sciences, University of Ioannina, Ioannina, Greece; 2 Department of Medicine, School of Health Sciences, University of Ioannina, Ioannina, Greece, University of Ioannina; 3 Intensive Care Unit, General Hospital of Ioannina, Ioannina, Greece

## Abstract

**Introduction:**

Discrepancies often arise between experts and non-experts regarding their roles in making end-of-life (EOL) decisions within the Intensive Care Unit (ICU). Such decisions are frequently contentious in clinical settings, with wide-ranging consequences in legal, ethical, psychological, social, and clinical contexts.

**Objectives:**

The aim of this study was to systematically review the global perspectives of physicians, nurses, family members, and the general public on who “should” be involved in decision-making for adult ICU patients, as well as to identify potential influencing factors.

**Methods:**

Adhering to the PRISMA 2020 guidelines, a comprehensive literature search was performed across PubMed, EMBASE, and CINAHL databases. A data extraction table was developed, validated through discussion and implemented by two independent researchers. The extracted data were subsequently analyzed descriptively.

**Results:**

Thirty-three studies were included, documenting variations in findings across different geographical and temporal contexts. Most participants in these studies were healthcare professionals. Despite evidence of paternalistic tendencies, physicians generally showed a growing inclination toward a more collaborative decision-making model. Similarly, the views of other population groups leaned towards patient and family involvement, with nurses additionally supporting their own participation. Six categories of influencing factors were identified, with legal/regulatory considerations and participant demographics emerging as the most significant.

**Conclusions:**

The overall representation of participants’ perceptions highlights a broader tendency towards collaborative decision-making. Τhis requires coordinated efforts from both clinical practitioners and policymakers to establish a decision-making framework that is inclusive, context-sensitive, and adaptable to the legal and cultural specifics of each region. To this end, emphasis should be placed on national-level interventions that address these issues directly, as opposed to broader, supranational approaches that may lack the necessary nuance.

**Disclosure of Interest:**

None Declared

